# Temperature-responsive peristome-structured smart surface for the unidirectional controllable motion of large droplets

**DOI:** 10.1038/s41378-023-00573-5

**Published:** 2023-09-28

**Authors:** Yunyun Song, Jialei Yang, Xu Zhang, Zhongqiang Zhang, Xinghao Hu, Guanggui Cheng, Yan Liu, Guojun Lv, Jianning Ding

**Affiliations:** 1 Institute of Intelligent Flexible Mechatronics, School of Mechanical Engineering, Jiangsu University, Zhenjiang, 212013 P. R. China; 2 State Key Laboratory of Structural Analysis for Industrial Equipment, Department of Engineering Mechanics, Faculty of Vehicle Engineering and Mechanics, Dalian University of Technology, Dalian, 116024 P. R. China; 3 Key Laboratory of Bionic Engineering (Ministry of Education), Jilin University, Changchun, 130022 P. R. China; 4 School of Environmental and Chemical Engineering, Jiangsu University of Science and Technology, Zhenjiang, 212003 P. R. China; 5 School of Mechanical Engineering, Yangzhou University, Yangzhou, 225127 Jiangsu P. R. China

**Keywords:** Microfluidics, Chemistry

## Abstract

The manipulation of fast, unidirectional motion for large droplets shows important applications in the fields of fog collection and biochemical reactions. However, driving large droplets (>5 μL) to move directionally and quickly remains challenging due to the nonnegligible volume force. Herein, we fabricated a scalable, bionic peristome substrate with a microcavity width of 180 μm using a 3D printing method, which could unidirectionally drive a large water droplet (~8 μL) at a speed reaching 12.5 mm/s by temperature-responsive wettability. The substrate surface was grafted with PNIPAAm, which could reversibly change its wettability in response to temperature, thereby enabling a temperature-responsive smart surface that could regulate droplet movement in real-time by changing the temperature. A series of temperature-responsive smart patterns were designed to induce water transport along specific paths to further realize controllable droplet motion with the antibacterial treatment of predesignated areas. The ability to achieve temperature-responsive unidirectional motion and dynamic control of droplet movement could allow programmable fluidic biosensors and precision medical devices.

**Figure:**
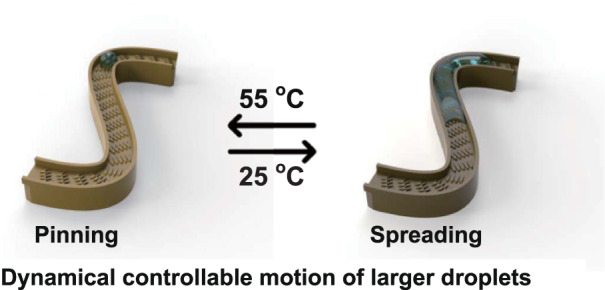
A temperature-responsive smart surface was produced to control the unidirectional motion of large droplets between spreading and pinning movement by changing the surface wettability.

A temperature-responsive smart surface was produced to control the unidirectional motion of large droplets between spreading and pinning movement by changing the surface wettability.

## Introduction

The unidirectional motion of droplets without external energy input has attracted much attention, and it has a wide range of applications, such as biomedical, controlled chemical reaction^1–3^, self-lubrication^4–6^, microfluidics^7,8^, and oil-water separation^9–12^. The bionic peristome surface of *Nepenthes alata* is a functional structural surface that has emerged in recent years, enabling droplets to spread continuously in a single direction under nondynamic conditions^13–15^. Due to the complex peristome structure of *Nepenthes alata*, scholars have used the bio-template method or ultraviolet (UV) lithography to reprint the peristome structure composed of anisotropic microgrooves and microcavities^16–19^. Moreover, UV lithography technology is complex and costly and is not suitable for mass production. Shang et al. used ferrofluid as a dynamic template to prepare a bionic surface with periodically aligned and overlapped arch-shaped microcavities to transfer droplets^20^. Geng et al. showed that the unidirectional motion of droplets can be achieved by drawing hydrophobic water barriers on a superhydrophilic 2D surface^21^. Nevertheless, the above studies are mainly based on the real size of the peristome structure of Nepenthes alata, and the structure is concentrated within a few tens of microns. The microcavity depth and scale of natural Nepenthes alata are too small (50 μm) to realize the unidirectional motion of large droplets (>5 μL) with fast velocities (>10 mm/s). Due to the nonnegligible volume force, it is difficult to drive large droplets to move directionally with fast velocities. The unidirectional motion of large droplets has important applications in the fields of fog collection, droplet manipulation, oil-water separation, biological and chemical reactions. Recently, integratable 3D printing and femtosecond laser technologies have been used to prepare complex micro/nanostructures. Researchers have combined 3D printing and templating methods to fabricate various patterns^22,23^. A phase-based spatially shaped femtosecond laser has been used to fabricate bionic surfaces with the unidirectional motion function of droplets^24^. These scholars have realized the unidirectional motion of large droplets, but the relationships between surface structure, water volume, and droplet movement remain unclear. The unidirectional motion of large droplets with fast velocities and long distances should be further pursued.

Controlling dynamic droplet movement on a functional surface plays a significant role in a wide range of applications, such as the autonomous spreading of fluids, the separation of liquid phases, the containment of backflow and the selective transmission of ions^25^. An external field can stimulate droplet movement. For example, Ren et al. switched the direction of fluid motion by simply shifting the position of the liquid metal droplets within the cylindrical chamber^26^. The scholars realized the large-scale separation of bioparticles in microchannels driven by AC dielectrophoresis^27^ and controlled two-phase flow in microfluidics using out-of-phase electro-convective streaming^28^. Changing the surface wettability is an effective method for controlling droplet movement. The change in the chemical components of smart materials under the stimulation of an external field leads to a change in their surface wettability. A smart peristome structure can be produced by grafting the temperature-responsive material PNIPAAm onto an artificial polydimethylsiloxane (PDMS) peristome substrate^29^; by changing the surface temperature, the authors have achieved the controllable movement of droplets^29^. A superamphiphilic organohydrogel with reconfigurable surface topography for programming the unidirectional motion of droplets has been developed, where highly adaptive wettability transitions between superhydrophilicity and superoleophilicity can be obtained^30^. A surface covered by tilted micro-cilia significantly controls droplet movement, including droplet adhesion and sliding, by regulating the external magnetic field, thus facilitating continuously and omnidirectionally controllable water delivery^31^. Although the abovementioned scholars have realized the smart control of droplet movement, due to the limitations of the preparation method and external field, it is difficult to achieve the unidirectional motion of droplets on complex patterns. Moreover, finding a method for dynamically controlling droplet movement and achieving the spreading and pinning of droplets on various patterns is still a great challenge.

In this study, we use the 3D printing method to produce a scaled-up bionic peristome substrate. By studying the effects of surface structure and water volume on motion properties, the surface achieves a unidirectional motion velocity reaching 12.5 mm/s with a large water volume of ~8 μL due to the extraordinary pinning-induced unidirectional motion and capillarity-induced ultrafast driving mechanisms. Moreover, a temperature-responsive smart surface is prepared by grafting poly(*N-isopropylacrylamide*) (PNIPAAm) onto the peristome substrate. Wettability and droplet movement are controlled by changing the surface temperature. The surface shows two different wettability states at various temperatures: hydrophobic above the lower critical solution temperature (LCST) and hydrophilic below the LCST. At 25 °C, the droplets spread in the positive direction and are pinned in the negative direction. At 55 °C, the droplets are pinned on the surface. Various patterns are designed to induce water transport along specific curved paths to further realize the antibacterial treatment of predesignated areas. The ability to achieve stable unidirectional motion of large droplets and dynamically control droplet movement provides the possibility for fog collection, programmable droplet manipulation, smart multiphase flow reactions and medical devices.

## Results and discussion

**Fig. 1:**
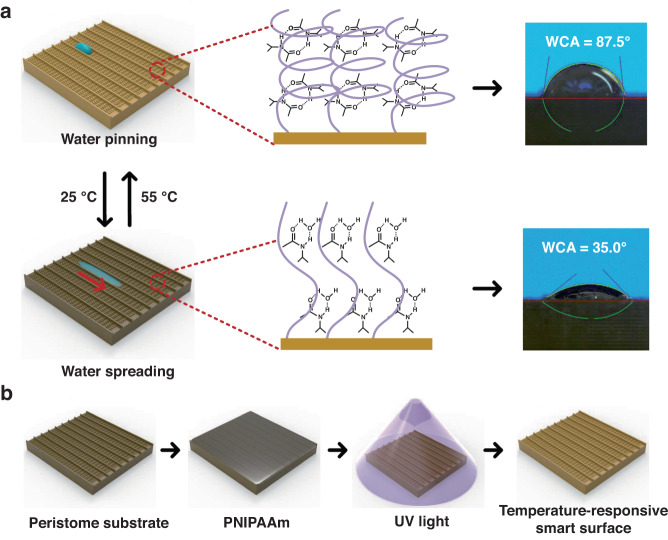
Design of a temperature-responsive smart surface. **a** Schematic diagram of the unidirectional spreading and pinning of droplets on the surface mediated by different temperatures. **b** Schematic diagram of the preparation process of the temperature-responsive smart surface

**Fig. 2:**
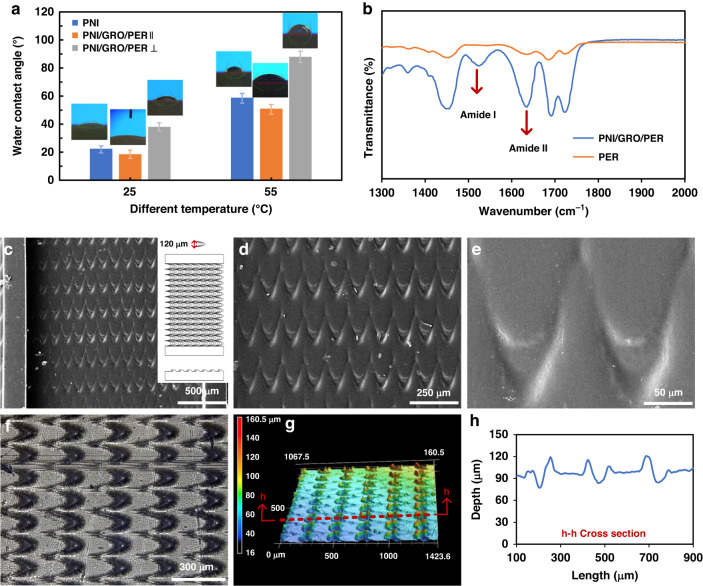
Wettability and surface morphology of the smart surface. **a** Wettability change in the PNI surface and PNI/GRO/PER surface in parallel and vertical microgroove directions at high and low temperatures. **b** FT-IR spectra of the PER substrate (yellow line) and the PNI/GRO/PER surface (blue line). **c**–**e** SEM images of the temperature-responsive smart surface at different magnifications. **f** 2D and (**g**) 3D optical images and (**h**) outline map of the microcavity structure on the smart surface from LCM

Herein, a temperature-responsive smart surface with unidirectional motion was prepared by grafting PNIPAAm onto the bionic peristome substrate of *Nepenthes alata*. As shown in Fig. 1a, the smart surface exhibits two different wettability states at distinct temperatures: hydrophilic below the LCST and hydrophobic above the LCST; this phenomenon occurs due to the reversible formation of intermolecular hydrogen bonding between PNIPAAm chains and water molecules (hydrophilic) below the LCST and intramolecular hydrogen bonding between C = O and N-H groups in PNIPAAm chains above the LCST (hydrophobic). At temperatures below 25 °C (below the LCST), the PNIPAAm surface with anisotropic peristome structures is hydrophilic, with droplets spreading in the positive direction and pinning in the negative direction. At temperatures above 55 °C (above the LCST), the surface is hydrophobic, with droplets being pinned on the bionic peristome surface. The fabrication of the peristome structure of Nepenthes alata is complicated by traditional two-step UV lithography and chemical etching methods. Herein, 3D printing technology can be applied to fabricate bionic peristome substrates in one step. Figure 1b presents the preparation process of the temperature-responsive smart surface. To prove that the wettability change is controlled by temperature, we measured the water contact angle (WCA) of the PNIPAAm surface (PNI), PNI surface with anisotropic microgroove and peristome structures (PNI/GRO/PER) at different temperatures (25 °C and 55 °C). As shown in Fig. 2a, at 25 °C, the WCAs of the PNI surface and the PNI/GRO/PER surface are small. The WCA in the parallel microgroove direction (PNI/GRO/PER∥) is smaller than that in the vertical microgroove direction (PNI/GRO/PER⊥) due to the anisotropic microgrooves. At 55 °C, the WCAs of both samples increase, where the WCA of the PNI/GRO/PER⊥surface reaches 87.5^°^. Thus, we realize a smart wettability change by controlling the temperature on the PNI/GRO/PER surface. The absorption peaks at approximately 1650 cm^−1^ of amide I and 1547 cm^−1^ of amide II indicate the successful modification of PNIPAAm on the bionic peristome substrate, as shown in Fig. 2b, which presents the Fourier transform infrared (FT-IR) spectra of the PNI/GRO/PER surface and the bionic peristome (PER) substrate.

The surface morphology of the smart surface, with anisotropic microgrooves and microcavities distributed along the microgrooves, is shown in Fig. 2c. The enlarged images (Fig. 2d, e) show that the microcavity width is approximately 120 μm, and the microcavity space along the microgroove is approximately 250 μm. The edge angle of the microcavity is approximately 30°. To further analyze the microcavity structure, we used laser confocal microscopy (LCM) to observe the surface morphology. Figure 2f shows the 2D optical image of the smart surface, where the microcavity structures are distributed anisotropically on the surface. The 3D optical image (Fig. 2g) and outline (Fig. 2h) of the microcavity structure show that the depth of the microcavity changes with a gradient, and the maximum value is approximately 25.86 μm.

**Fig. 3:**
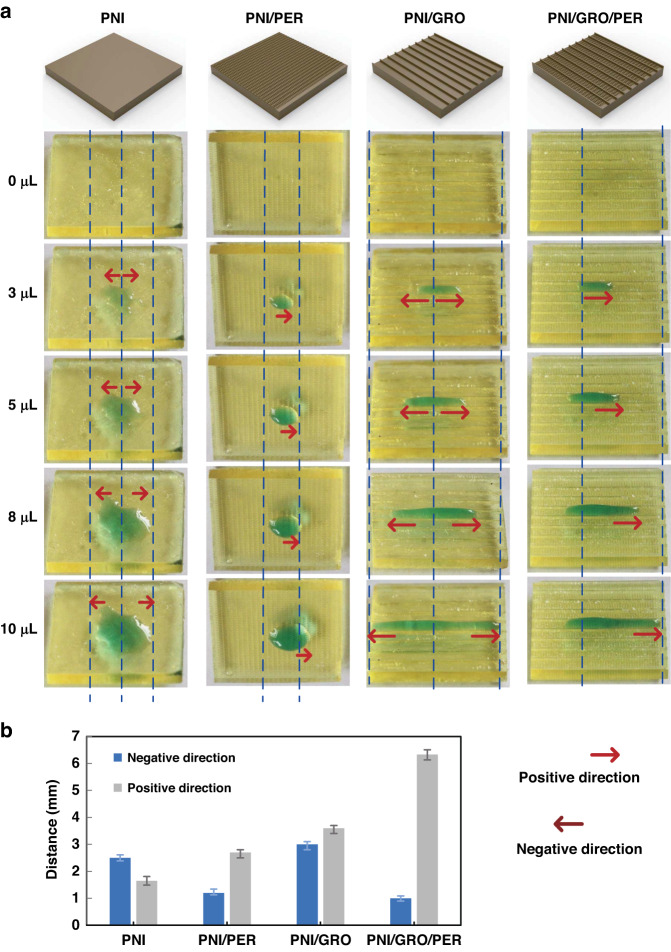
Droplet movement with different volumes on various surfaces (PNI surface, PNI/PER surface, PNI/GRO surface and PNI/GRO/PER surface). **a** Optical microscopy images of the detailed process showing water spreading on various surfaces with different water volumes. **b** Comparison of the spreading distance of 5 μL water on various surfaces in the positive direction and negative direction

To prove the unidirectional motion of the PNI/GRO/PER surface, we analyzed droplet movement with different volumes on various surfaces, including the PNI surface, the PNI surface with peristome structure (PNI/PER), the PNI surface with anisotropic microgrooves (PNI/GRO), and the PNI/GRO/PER surface; the specific process of droplet movement was recorded by a camera. Figure 3a shows droplet movement with different volumes on various surfaces. With increasing volume, water spreads evenly on the PNI surface due to its hydrophilicity. On the PNI/PER surface, water spreads in one (positive) direction and is pinned in another (negative) direction. However, the spreading distance is short due to the lack of anisotropic microgrooves. The PNI/GRO/PER surface shows obvious unidirectional motion due to the anisotropic microgrooves and peristome structure. Due to the absence of the peristome structure, water spreads the same distance in the positive direction and negative direction on the PNI/GRO surface, where water is limited in microgrooves. To quantitatively compare the unidirectional motion of different surfaces, we counted the spreading distances of 5 μL water in both directions of various specimens in Fig. 3b. Based on the above analysis, we conclude that the anisotropic microgrooves and peristome structure are two essential factors for realizing unidirectional motion. The special unidirectional motion behavior of droplets is attributed to the combination of the hydrophilic PNI surface and the capillary force inside the microcavity.

**Fig. 4:**
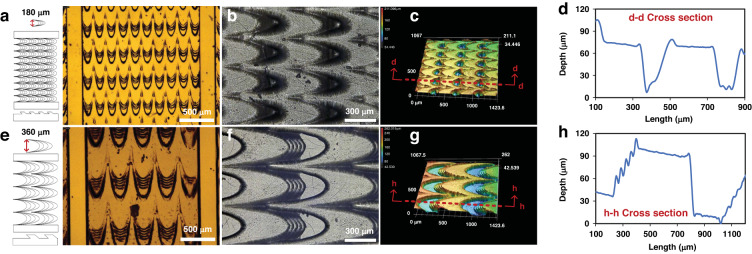
Surface morphologies and 3D optical images of the 180 and 360 surfaces. **a**, **e** PNI/GRO/PER surface images with microcavity widths of 180 μm and 360 μm under optical microscopy, respectively. **b**, **f** 2D optical images. **c**, **g** 3D optical images. **d**, **h** Outline maps of the microcavity structures on the 180 and 360 surfaces from LCM, respectively

Traditional studies have mainly been based on the real size of the peristome structure of *Nepenthes alata*; the size of the peristome structure was considered approximately a few tens of microns. However, the structure size affects droplet movement. Moreover, the relationships between peristome structure, water volume and droplet movement remain unclear. Revealing the relationship between them will greatly expand the applicability of the bionic peristome structure. By analyzing the peristome structure of *Nepenthes alata*, we designed three surfaces with different microcavity sizes according to their fluid-driven mechanisms. The microcavity wedge angle of the different peristome structures is 30°, and the microcavity widths are 120 μm, 180 μm, and 360 μm; the three samples have been simplified and named 120, 180, and 360, respectively. Figure S1 shows the model diagrams of the 120, 180, and 360 surfaces, where they maintain a microgroove width of approximately 1730 μm. Figure 4a, e shows the images of the 180 and 360 surfaces taken by an inverted microscope (IM), respectively. Despite the layer height error and step error in 3D printing, the overall wedge structures and microcavities were perfectly prepared to form a multiscale array structure. To further analyze the microcavity structures of the 180 and 360 surfaces, we used LCM to observe the surface morphologies. Figure 4b, f shows the 2D optical images of the 180 and 360 surfaces, where the microcavity structures are distributed anisotropically on the surface. The microcavity width of the 180 surface is approximately 180 μm, and the microcavity space along the microgroove is approximately 380 μm. The microcavity width of the 360 surface is approximately 360 μm, and the microcavity space along the microgroove is approximately 760 μm. The 3D optical images (Fig. 4c, g) and outline map (Fig. 4d, h) of the microcavity structures show that the depth of the microcavity changes with a gradient, and the maximum values of the 180 and 360 surfaces are approximately 65.14 μm and 105.93 μm, respectively. Additionally, the optical images of the 180 and 360 surfaces from the superdepth field microscope (SFM) are illustrated in Fig. S2, and the findings are consistent with the above analysis.

**Fig. 5:**
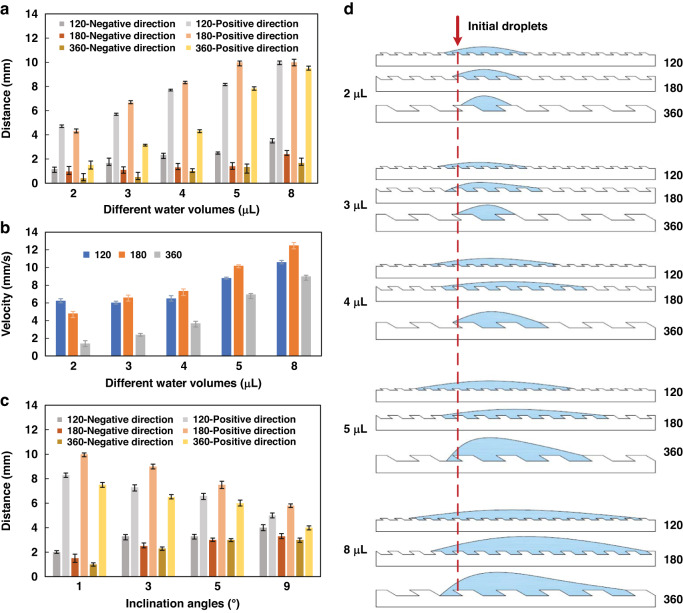
Spreading distances and velocities of different water volumes on various surfaces in both directions. **a** Comparison of the spreading distances of different water volumes on various surfaces in the positive direction and negative direction. **b** Comparison of the spreading velocities of different water volumes on various surfaces in the positive direction. **c** Spreading distances of 5 μL water on various surfaces in the positive direction and negative direction at different tilt angles. **d** Cross-sectional view of droplet movement of different volumes on various surfaces

To further clarify the effect of the peristome structure on droplet movement, we compared the spreading distances of the various surfaces in both directions. As shown in Fig. 5a, the 120, 180, and 360 surfaces show obvious anisotropic properties; with the increase in the volume of droplets, the spreading distance increases. However, for the same water volume, the spreading distances of the 120 and 180 surfaces are longer than that of the 360 surfaces in the positive direction because the small microcavity depths of the 120 and 180 surfaces are advantageous to droplet movement, which shows larger capillarity. Moreover, the 180 and 360 surfaces show obvious anisotropic properties, especially for the large droplet. This phenomenon occurs because the rough peristome structure inhibits the water from spreading in the negative direction due to the obvious pinning effect. When the water volume exceeds 5 μL, it is difficult for the 120 surface to obstruct water spreading in the negative direction due to the reduced pinning effect of the small microcavity depth. Thus, when the water volume is less than 5 μL, the 120 and 180 surfaces can realize unidirectional motion over a long distance; when the water volume exceeds 5 μL, the 180 and 360 surfaces can realize obvious unidirectional motion.

In addition to the water spreading distance, we measured the spreading velocity in Fig. 5b. Consistent with the above analysis, with increasing water volume, the spreading velocity increases. For the same water volume, the spreading velocities of the 120 and 180 surfaces are faster than that of the 360 surface due to the enhanced capillarity of small peristome structures and microcavity depths. However, with the increase in water volume, the spreading velocity of the 180 surface is faster than that of the 120 surface. The small peristome structure and microcavity depth cannot block the water from spreading in the negative direction, which causes a short spreading distance in the positive direction. Herein, the 180 surface achieves the fastest spreading velocity of approximately 12.5 mm/s.

To further prove the unidirectional motion of droplets, we adjusted the tilt angle of the test platform to measure the spreading distances of 5 μL water on various surfaces in both directions at different tilt angles, as shown in Fig. 5c. The results show that the droplets can still be transported along the track at different tilt angles. With increasing tilt angle, the spreading distance in the positive direction first gradually decreases and then increases in the negative direction. This phenomenon suggests that the surface can transport droplets against gravity. When the tilt angle exceeds 5°, the 120 surface misses obvious unidirectional motion, where the large peristome structure and microcavity depth can overcome gravity and hinder water spreading in the negative direction. Thus, the 180 and 360 surfaces can realize the excellent unidirectional motion of large droplets.

According to Fig. 5a, a schematic diagram of the spreading distance on various surfaces in both directions was drawn (Fig. 5d), where the red dashed line indicates the position of the initial droplet. When the water volume is less than 5 μL, the 120 surface can realize excellent unidirectional motion over a long distance. When the water volume exceeds 5 μL, the smaller peristome structure cannot block droplet movement in the negative direction. The 180 and 360 surfaces can realize unidirectional motion of 5 μL water. The large peristome structure blocks water transport in the negative direction and limits droplet movement in the positive direction. Thus, to achieve fast water transport, we used the 180 surface as the experimental subject.

**Fig. 6:**
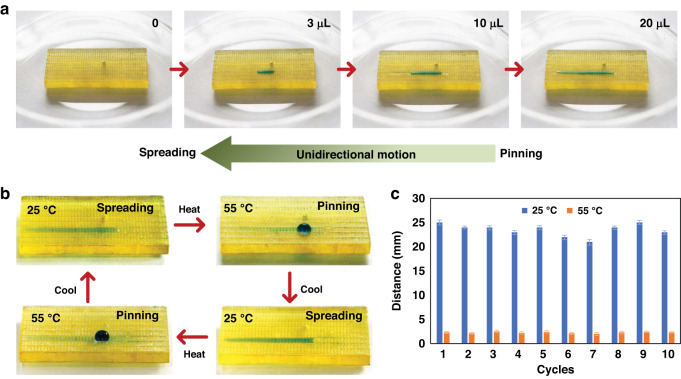
Observation of droplet movement on the PNI/GRO/PER surface at different temperatures. **a** As the droplet volume continues to increase, the droplet spreads unidirectionally on the surface. **b** The PNI/GRO/PER surface allows for reversible unidirectional spreading and pinning of droplets when the temperature is changed. **c** Spreading distances of the droplets at different temperatures over 10 cycles

The PNI/GRO/PER surface was repeatedly heated and cooled between 55 °C and 25 °C to change the wettability and droplet movement. At 25 °C, the PNIPAAm surface is hydrophilic, with droplets spreading in the positive direction and pinning in the negative direction on the PNI/GRO/PER surface. At 55 °C, the PNIPAAm surface is hydrophobic, with droplets being pinned on the PNI/GRO/PER surface. Figure 6a shows the unidirectional motion process of droplets at 25 °C. The droplets stained by methylene blue were continuously dropped onto the PNI/GRO/PER surface. With the increase in droplet volume, there is an obvious tendency for the droplets to move to the left and pin in the opposite direction, showing obvious unidirectional motion characteristics.

Figure 6b shows the change in the droplet movement state at different temperatures, where the droplets first spread unidirectionally when the temperature is below the LCST (25 °C) and are then pinned to the PNI/GRO/PER surface when the temperature is above the LCST (55 °C). At 25 °C, when the water volume is 20 μL, the spreading distance reaches 23.4 mm. After repeatedly heating and cooling the PNI/GRO/PER surface 10 times, the surface exhibits excellent reversibility and stability for its application. Although the unidirectional motion of droplets has been achieved in some previous studies, it is difficult to achieve dynamic control of the droplet movement state. The PNI/GRO/PER surface can achieve unidirectional spreading at 25 °C and pinning at 55 °C. The PNI/GRO/PER surface can control droplet movement by temperature.

**Fig. 7:**
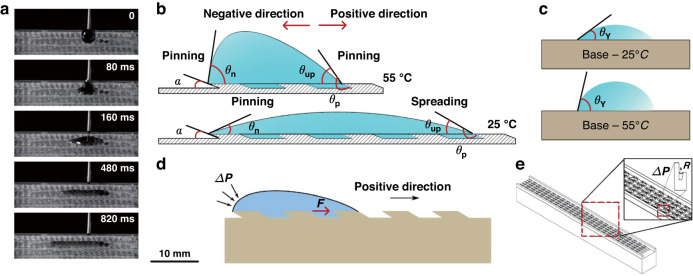
Mechanism of unidirectional motion of droplets on the PNI/GRO/PER surface **a** Unidirectional motion of 5 μL water on the 180 surface. **b** Wetting state of droplets at high and low temperatures. **c** ${\theta }_{{\rm{Y}}}$ is the intrinsic WCA of the PNI surface at different temperatures. **d** Force analysis of droplets during unidirectional motion. **e** Diagram of the microstructure in a single track

The unidirectional motion mechanism was explored by studying the wetting mechanisms at different temperatures. Figure 7a and Video S1 show the unidirectional motion of 5 μL water on the 180 surface through a high-speed camera, where the droplets move in the positive direction over a long distance and move in the negative direction over a short distance. Figure 7b shows a diagram of the wetting states of droplets at different temperatures. At 25 °C, the wetting process of droplets on the PNI/GRO/PER surface is continuous and periodic; when the first microcavity has not been completely wetted, the droplets have already entered the next microcavity. We investigated the edge effect of the microcavity on water spreading, and the unidirectional motion of droplets on the PNI/GRO/PER surface was jointly determined by the forward spreading distance and the reverse spreading distance. In the negative direction, due to the presence of elliptical sharp edges, the reverse spreading of droplets is inhibited, and the pinning effect occurs. Liu’s model states that the pinning effect occurs when the contact angle $\theta$ satisfies the following equation^32^:

1 $${\theta }_{Y}\le \theta \le {\theta }_{{\rm{Y}}}+\pi -\alpha$$

where $\theta$ is the contact angle between the droplet and the substrate, $\alpha$ is the microcavity wedge angle of the peristome structures ($\alpha ={30}^\circ$), and ${\theta }_{{\rm{Y}}}$ is the intrinsic WCA of the PNI surface.

The advanced angle of the droplet on the horizontal line is defined as ${\theta }_{{\rm{up}}}$, and ${\theta }_{{\rm{p}}}$ and ${\theta }_{{\rm{n}}}$ are the advanced angle and receding angle of the droplet on the peristome structure, respectively. The relationship between ${\theta }_{{\rm{up}}}$ and ${\theta }_{{\rm{p}}}$ is as follows:

2 $${\theta }_{{\rm{p}}}= (180^\circ \,-\alpha)+{\theta }_{{\rm{up}}}$$

We know that the condition of the water spreading over the sharp edge is ${\theta }_{{\rm{p}}}/{\theta }_{{\rm{n}}}$ > ${\theta }_{{\rm{Y}}}+\pi -\alpha$. Herein, ${\theta }_{{\rm{p}}}$ is much greater than ${\theta }_{{\rm{n}}}$. By comparing ${\theta }_{n}$ with ${\theta }_{{\rm{p}}}$, it is found that ${\theta }_{{\rm{p}}}$ easily exceeds the critical value; thus, the droplet tends to diffuse across the sharp edge in the positive direction. After the droplet diffuses over the sharp edge of the ellipse, under the action of capillary force, the droplet diffuses inward from both sides of the microcavity^16^ and finally fills the whole microcavity and diffuses to the edge of the ellipse. Regardless of the surface wettability, the droplets in the negative direction can be easily pinned at the sharp edge of the ellipse.

On the PNI/GRO/PER surface, the spreading distance increases as ${\theta }_{{\rm{up}}}$ decreases. When ${\theta }_{{\rm{up}}}\,>\,{\theta }_{{\rm{Y}}}$, to realize the wettability balance, the droplet spreads in the positive direction; when ${\theta }_{{\rm{up}}}$ < ${\theta }_{{\rm{Y}}}$, the droplet is pinned on the surface. The PNI surface exhibits different wettability levels at various temperatures. As shown in Fig. 7c, at 25 °C, the intrinsic contact angle *θ*_Y_ is approximately 23°; at 55 °C, *θ*_Y_ is approximately 58°. When the surface is highly hydrophilic, ${\theta }_{{\rm{up}}}$ is small, and the spreading distance increases. When the surface hydrophilicity weakens, ${\theta }_{{\rm{up}}}$ is large, and the spreading distance decreases. Thus, the PNI/GRO/PER surface shows a change in droplet movement from spreading to a pinning state when the temperature increases from ~25 °C to ~55 °C, and the change in movement behavior is reversible.

Additionally, we performed a force analysis for the droplet during droplet movement. In the negative direction, the droplets appear as semicircular arcs, where the surface tension of the water and geometric structure lead to the Laplace pressure $\varDelta p$ at the gas‒liquid interface. According to the famous Young–Laplace equation^33^, $\varDelta p={\rm{\gamma }}\left(\frac{1}{{R}_{1}}+\frac{1}{{R}_{2}}\right)\,$, where $\varDelta p$ is the Laplace pressure difference on both sides of the droplet interface at any point^34^, $\gamma$ is the surface tension coefficient of the droplet, and ${R}_{1}$ and ${R}_{2}$ are the radii of curvature at any point on the contact surface between the droplet and the gas. When the contact surface is spherical (${R}_{1}={R}_{2}$), $\varDelta p$ can be expressed as $\varDelta p=\frac{2{\rm{\gamma }}}{R}$.

As shown in Fig. 7d, the Laplace pressure $\varDelta p$ inhibits droplet movement in the negative direction. Wettability affects the radius of curvature of the contact surface. At 25 °C, the WCA of the PNI/GRO/PER surface is approximately 37°; at 55 °C, the WCA is approximately 88°. When the surface is highly hydrophilic, most water spreads in the positive direction, and little water is pinned in the negative direction, increasing the radius of curvature. When the surface hydrophilicity weakens, most water is pinned in the negative direction, reducing the radius of curvature. Thus, at 25 °C, the resistance in the negative direction is relatively small, and at 55 °C, the resistance in the negative direction is relatively large. In the positive direction, the capillary force is the main driving force for droplet movement.

The diagram of the microstructure in a single track is shown in Fig. 7e. The gap between the sidewall and the microstructure is another reason for pinning the droplets in the negative direction. According to the Young–Laplace equation, due to the surface tension of the droplet at the gap, the radius of curvature *R* increases with increasing water column height, and $\varDelta p$ decreases to pin the droplet. Based on the above result, we noted the different droplet movement behaviors of the 120, 180, and 360 surfaces. Figure S3 shows the WCAs of the various surfaces, including the peristome substrates (GRO/PER surface) and PNI/GRO/PER surface, where the WCAs of the 120, 180, and 360 surfaces increase in sequence regardless of the PNI surface. According to Formula $\varDelta p=\frac{2{\rm{\gamma }}}{R}$, a greater WCA leads to a smaller radius of curvature *R* and a larger Laplace pressure $\varDelta p$. There is more resistance for the droplet to spread in the negative direction for the 180 and 360 surfaces than for the other surfaces. According to Formula (1), a small ${\theta }_{{\rm{up}}}$ increases the spreading distance. The 120 surface has a long spreading distance. Thus, the 180 and 360 surfaces show obvious anisotropic properties, especially for large droplets, due to the obvious pinning effects of their large peristome structures.

**Fig. 8:**
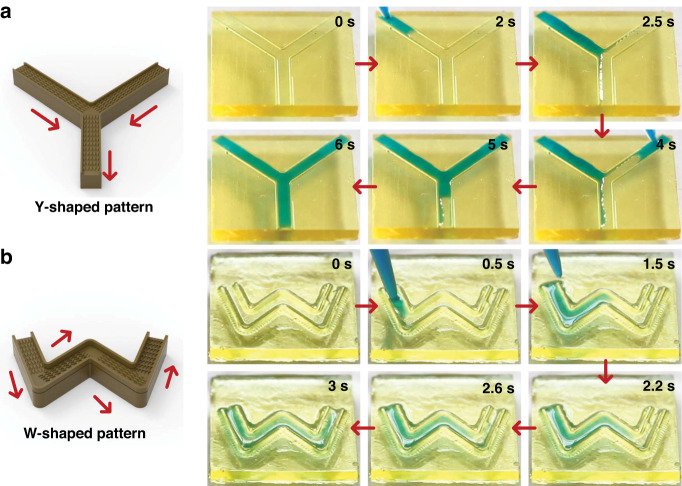
Unidirectional motion of droplets on different linear patterns. **a**, **b** Unidirectional motion of droplets in Y- and W-shaped patterns

Although the unidirectional motion of droplets has been achieved in some previous studies, achieving the unidirectional motion of droplets on various patterns with a topography structure is still a difficult problem to solve, which can provide new possibilities and options for smart microfluidic systems. Assisted by 3D printing technology, we designed two kinds of linear patterned peristome substrates—a Y-shaped pattern and a W-shaped pattern—with different orientations. As shown in Fig. 8a, the Y-shaped pattern is composed of three linear tracks with different orientations, and the directions of different orientations in the Y-shaped pattern are shown by arrows. A 20 μL droplet was added to the head edges of both patterns; the droplets spread unidirectionally along the different orientations and eventually converge on the third track, providing a new idea for microchemical reactors. The unidirectional motion of droplets on the Y-shaped pattern is shown in Video S2 in the Supporting Information. In addition to the continuous spreading on the Y-shaped pattern with a single bending structure, Fig. 8b shows that the droplets can spread unidirectionally on the W-shaped pattern with multiple bending structures. Continuously deposited large droplets can be transported from the left to the right along the pathways of the W-shaped pattern. The unidirectional motion of droplets on the W-shaped pattern is shown in Video S3 in the Supporting Information.

**Fig. 9:**
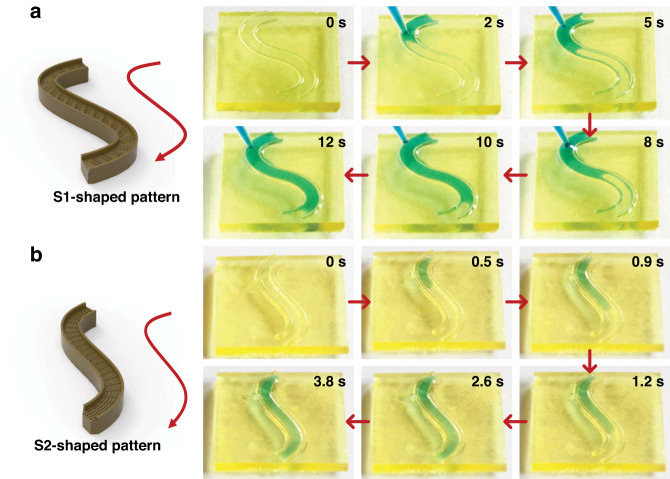
Unidirectional motion of droplets on different curvilinear patterns. **a**, **b** Unidirectional motion of droplets in different curved S-shaped patterns

**Fig. 10:**
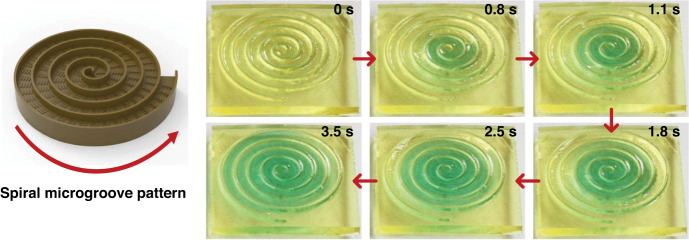
Unidirectional motion of droplets on a spiral microgroove pattern

In addition to the linear patterns, we designed curved peristome substrates: S1-shaped pattern, S2-shaped pattern (S1 and S2 represent curved patterns with different curvatures), and spiral microgroove pattern with different orientations. Figure 9 shows that the droplets can spread unidirectionally along the S-shaped patterns, where the spreading velocity of the S2-shaped pattern is faster than that of the S1-shaped pattern. The unidirectional motion of droplets on the S-shaped patterns is shown in Videos S4 and S5 in the Supporting Information. To further prove the unidirectional motion of a pattern with a larger curvature, we designed a spiral microgroove pattern with an anticlockwise orientation. As shown in Fig. 10, continuously deposited droplets can be transported along the pathways of the spiral microgroove pattern in the anticlockwise orientation. The unidirectional motion of droplets on the spiral microgroove pattern is shown in Video S6 in the Supporting Information. Additionally, we designed an alphabetic University Jiangsu (UJS)-shaped pattern and recorded the unidirectional motion process, as shown in Fig. S4 and Video S7. Unlike in linear patterns, in curved patterns, the curvature alteration generates a Laplace pressure difference, and the larger curvature causes a larger Laplace pressure difference and a higher velocity. However, the spreading velocity of the S2-shaped pattern (small curvature) is faster than that of the S1-shaped pattern (large curvature). This phenomenon occurs because the motion velocity of droplets on the concave side is larger than that on the convex side of the same curved patterns^35^. For the S1-shaped pattern, the convex side greatly inhibits the unidirectional motion of droplets. The complex patterns based on such a topologically peristome substrate open an avenue to achieve accurate liquid transport control and explore potential applications in food processing, moisture transfer, heat management, and inkjet printing processes.

**Fig. 11:**
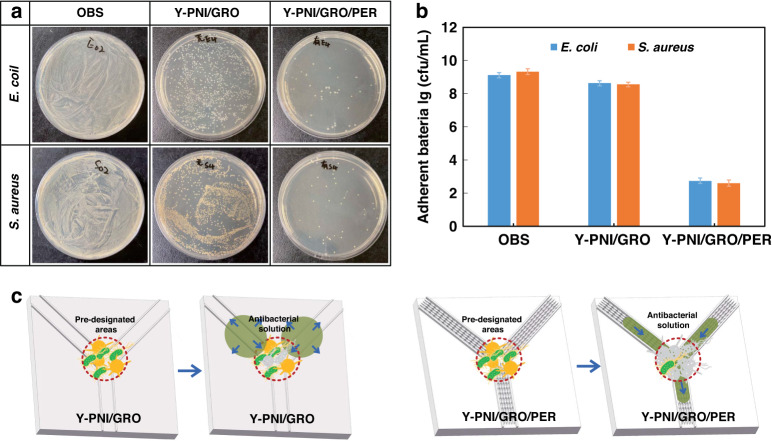
The antibacterial treatment of predesignated areas on the Y-shaped pattern. **a** Photograph of bacterial colonies (original bacterial suspension: OBS, Y-shaped pattern with microgroove structures: Y-PNI/GRO, and Y-shaped pattern with microgroove and peristome structures: Y-PNI/GRO/PER) growing on predesignated areas of different samples. **b** Statistical amounts of adherent bacteria on the predesignated areas of different samples by the plate counting method. **c** Schematic diagram of the antibacterial processes on the Y-PNI/GRO and Y-PNI/GRO/PER patterns

The complex patterns can achieve the antibacterial treatment of predesignated areas. By taking the Y-shaped pattern as an example, we compared the antibacterial property of the Y-shaped pattern with microgroove and peristome structures (Y-PNI/GRO/PER) with that of the Y-shaped pattern with microgroove structures (Y-PNI/GRO) in Fig. 11. As shown in Fig. 11c, we inoculated gram-negative Escherichia coli (*E. coli* ATCC 25922) and gram-positive Staphylococcus aureus (*S. aureus* ATCC 6538) at the center of the Y-shaped patterns. Then, a droplet of 20 μL antibacterial solution containing tea polyphenols was continuously added to the head edges of both tracks, and the droplets moved unidirectionally along the different orientations and eventually converged on the center of the Y-PNI/GRO/PER pattern, where the tea polyphenols killed the adherent bacteria. Due to the lack of peristome structure, on the Y-PNI/GRO pattern, the droplets would move out of the microgroove due to the greater inertia from a large amount of water, and it was difficult to kill the bacteria. Figure 11a shows a photograph of bacterial colonies from the Y-PNI/GRO and Y-PNI/GRO/PER patterns after antibacterial treatment. The adhered bacteria were eluted by thoroughly washing the samples in tubes containing 5 mL 0.85% NaCl solution. The obtained suspensions were diluted by the 10-fold dilution method and spread onto an LB agar plate (100 μL). Finally, the plates were used to enumerate the number of adherent bacteria after incubation at 37 °C for 24 h (Fig. 11b). The result shows that the Y-PNI/GRO/PER track can effectively guide the unidirectional motion of the antibacterial solution to the predesignated area to kill the bacteria. Compared with the Y-PNI/GRO pattern, the Y-PNI/GRO/PER pattern can reduce the bacterial adhesion rate by 75%.

## Conclusion

In this study, inspired by the unidirectional liquid diffusion of Nepenthes alata, we used 3D printing to produce a peristome-mimetic substrate. We studied the relationships between the peristome structure, water volume and droplet movement. When the water volume was less than 5 μL, the 120 surface could realize excellent unidirectional motion over a long distance. When the water volume exceeded 5 μL, the small peristome structure could not block the droplet movement in the negative direction, and the 180 and 360 surfaces could realize the unidirectional motion of 5 μL water. The large peristome structure blocked droplet movement in both directions. Thus, by adjusting the surface structures, the 180 surface achieved the unidirectional motion of large droplets with a velocity of 12.5 mm/s, water volume of 20 μL, and distance of 23.4 mm. Herein, a temperature-responsive smart surface was prepared by grating PNIPAAm onto the substrate. The wettability and droplet movement were controlled by changing the surface temperature. At 25 °C, droplets spread in the positive direction and were pinned in the negative direction. At 55 °C, the droplets were pinned on the surface. A series of temperature-responsive smart patterns (Y, S, W, spiral, and alphabetic) were designed to induce water transport along specific curved paths to further realize the antibacterial treatment of predesignated areas. The abilities to achieve stable unidirectional motion and to dynamically control droplet movement provided the possibility for fog collection, programmable droplet manipulation, oil-water separation, and medical devices.

## Experimental section

### Materials

N-Isopropylacrylamide (NIPAM, ≥98%) and N,N-methylenebisacrylamide bis-acrylamide (MBA, ≥99%) were purchased from Shanghai Macklin Biochemical Co., Ltd. Benzophenone (≥99%), methylene blue (≥90%) and benzoin ethyl ether (≥97%) were purchased from 809 Chuhua Branch Road, Fengxian District, Shanghai. Photoreactive resin UTL was purchased from BMF Precision Tech Inc. Deionized water was obtained from a UNIQUE-R10 system.

### Fabrication of bionic peristome substrate

Three bionic peristome substrates with different scales of peristome structures were designed by CAD software, where the microcavity widths were 120 μm, 180 μm, and 360 μm. The equipment used to prepare the peristome structure and microgroove was a Micronano 3D Printer (nanoArch S140, Shenzhen, China), and the minimum exposure thickness is 10 μm. The printed material was UTL (low-viscosity super tough resin). The prepared substrates were ultrasonically cleaned in alcohol for 5 min. The samples were then put into a blast drying oven at 40 °C for 1 h to dry.

### Fabrication of the PNI/GRO/PER surface

We mixed NIPAM with MBA in a ratio of 200:1 and added an appropriate amount of deionized water, which was then stirred continuously for 2 h at 60 °C for full dissolution. Nitrogen gas was passed to remove the oxygen in the device to obtain the NIPAM solution. In the experiment, first, the bionic peristome substrate was treated with plasma for 10 min to produce sufficient hydroxyl groups. Second, the bionic peristome substrate was washed with deionized water for 3 min and immersed in 20 wt% benzophenone + acetone for 10 min. The photoinitiator (benzophenone) was attached to the substrate surface and produced reaction sites. The substrate was washed with deionized water for 5 min and soaked in NIPAM solution for 2 min. The substrate was rotated at 300 rpm for 10 s. Then, the substrate was irradiated with UV light emitted from a high-pressure mercury lamp at approximately 60 cm from the lamp for 6 min. As a result, NIPAM molecules formed covalent bonds on the previously generated reaction sites and formed a thin film on the substrate. After the reaction was completed, the substrate was sequentially rinsed with deionized water and acetone for 2 min to remove the excess NIPAM.

### Instrumentation

The surface morphologies of the different structures were photographed with a scanning electron microscope (SEM, EVO 18, ZEISS), ultradepth microscope (UDM, DSX 510, OLYMPUS), and inverted microscope (IM, IX73, OLYMPUS). The elemental composition of the surface was analyzed by Fourier transform near-infrared spectroscopy (FT-IR, FTIR-4100, JASCO). The unidirectional motion characteristics of droplets on different patterns were recorded by a camera (Canon 80D, Japan) with a timescale. WCA was measured by an OCA20 machine (Data-Physics, Germany) for various surfaces at different temperatures.

### Supplementary information

Supporting Information

Video S1

Video S2

Video S3

Video S4

Video S5

Video S6

Video S7
