# Supplementary material for: Temperature-responsive peristome-structured smart surface for the unidirectional controllable motion of large droplets
Source: Microsyst Nanoeng. 2023 Sep 28;9:119. doi: 10.1038/s41378-023-00573-5 (PMC10539527; doi:10.1038/s41378-023-00573-5)
Supplement: Supplementary file 1 — Supporting Information [file 41378_2023_573_MOESM1_ESM.docx]

**Supporting Information**

Temperature-responsive peristome-structured smart surface for the unidirectional controllable motion of large droplets

Yunyun Song^a^, Jialei Yang^a^, Xu Zhang^a^, Zhongqiang Zhang^a, b^*, Xinghao Hu^a^*, Guanggui Cheng^a^, Yan Liu^c^, Guojun Lv^d^, Jianning Ding^a, e^*

^a^. Institute of Intelligent Flexible Mechatronics, School of Mechanical Engineering, Jiangsu University, Zhenjiang 212013, P. R. China

^b^. State Key Laboratory of Structural Analysis for Industrial Equipment, Department of Engineering Mechanics, Faculty of Vehicle Engineering and Mechanics, Dalian University of Technology, Dalian, 116024, P. R. China

^c^. Key Laboratory of Bionic Engineering (Ministry of Education), Jilin University, Changchun, 130022, P. R. China

^d^. School of Environmental and Chemical Engineering, Jiangsu University of Science and Technology, Zhenjiang, 212003, P. R. China

^e^. School of Mechanical Engineering, Yangzhou University, Yangzhou, 225127,

Jiangsu, P. R. China

*E-mail: [zhangzq@ujs.edu.cn](mailto:zhangzq@ujs.edu.cn); huxh@ujs.edu.cn; dingjn@ujs.edu.cn


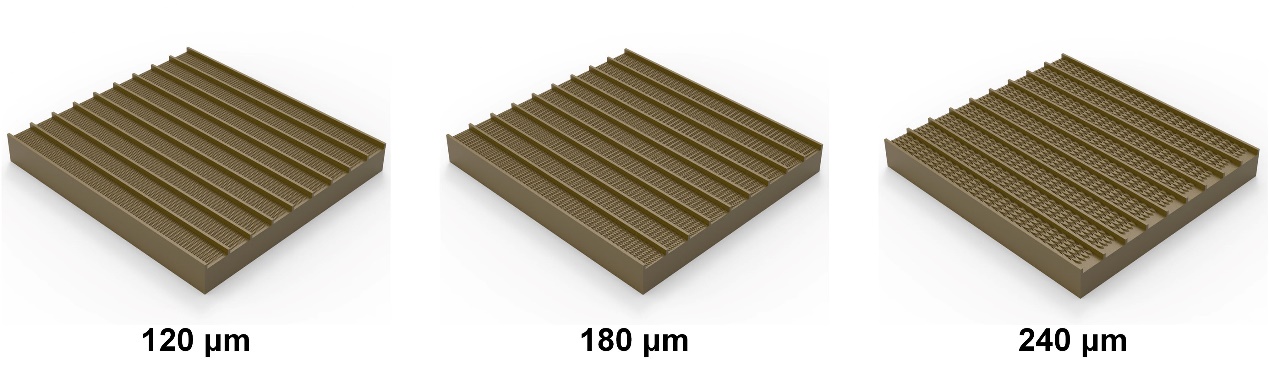


**Figure S1.** The model diagrams of the 120, 180 and 360 surfaces, they maintain a microgroove width of approximately 1730 μm.


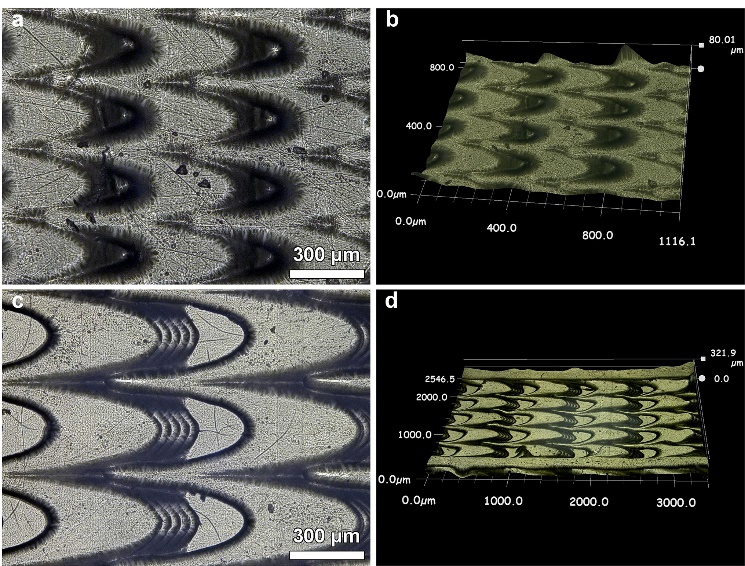


**Figure S2.** The optical images of the (a-b) 180 and (c-d) 360 surfaces from the SFM.


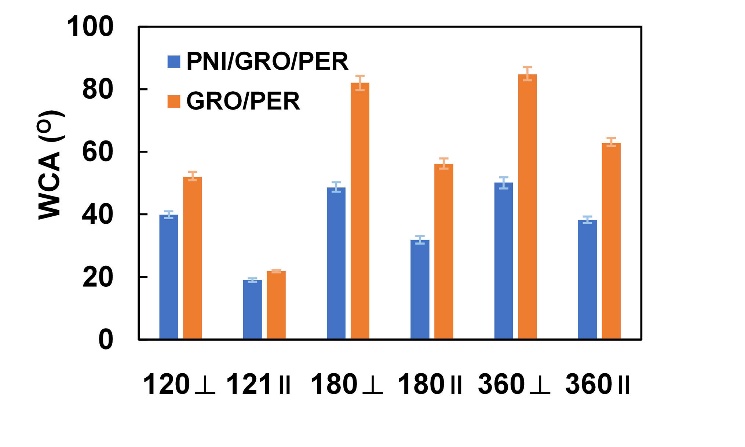


**Figure S3**. The WCA of the various surfaces including peristome substrates (GRO/PER surface) and resin substrates modified by PNI (PNI/GRO/PER surface).


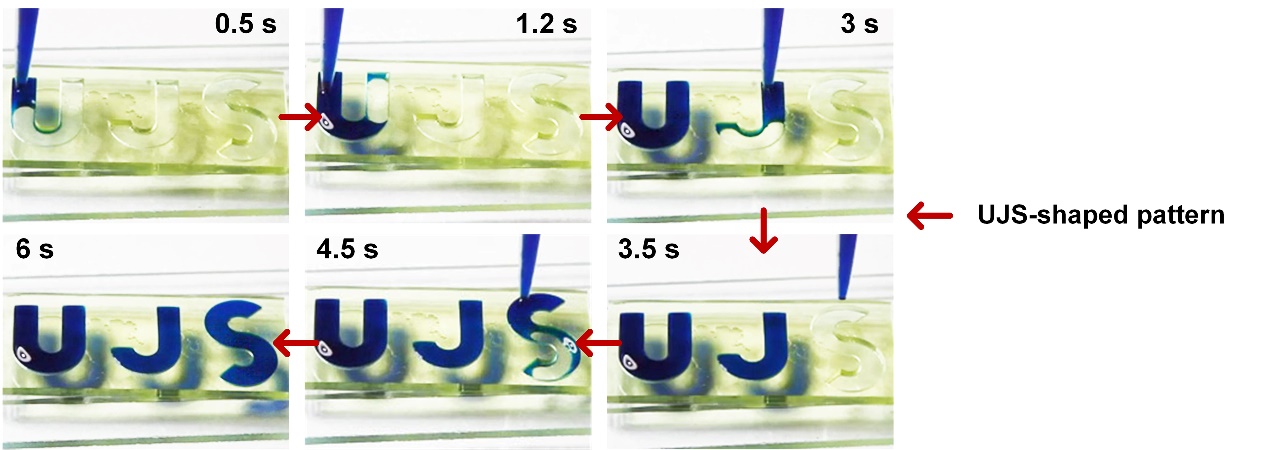


**Figure S4**. Unidirectional motion of droplets on the UJS-shaped pattern.

**Video S1**. The unidirectional motion of 5 μL water on the 180 surface.

**Video S2**. The unidirectional motion of droplets on the Y-shaped pattern.

**Video S3**. The unidirectional motion of droplets on the W-shaped pattern.

**Video S4**. The unidirectional motion of droplets on the S1-shaped pattern.

**Video S5**. The unidirectional motion of droplets on the S2-shaped pattern.

**Video S6**. The unidirectional motion of droplets on the spiral microgroove pattern.

**Video S7**. The unidirectional motion of droplets on the UJS-shaped pattern.
